# Non-Uptake of Newborn Screening in Planned Homebirth Is Associated with Preventive Health Practices for Infants: A Retrospective Case-Control Study

**DOI:** 10.3390/ijns11010015

**Published:** 2025-02-21

**Authors:** Chen Stein-Zamir, Hanna Shoob, Sandra Katan, Gina Verbov, Shlomo Almashanu

**Affiliations:** 1 Faculty of Medicine, Braun School of Public Health and Community Medicine, The Hebrew University of Jerusalem, Jerusalem 9112102, Israel; gina.leib@lbjr.health.gov.il; 2 Jerusalem District Health Office, Ministry of Health, Jerusalem 9134302, Israel; hanna.shoob@lbjr.health.gov.il (H.S.); sandra.katan@moh.gov.il (S.K.); 3 National Newborn Screening Program, Ministry of Health, Ramat Gan 5262000, Israel; shlomo.almashanu@sheba.health.gov.il

**Keywords:** newborn screening, newborn, infant, planned homebirth, hospital birth, mother and child health clinic, childhood immunizations

## Abstract

Universal Newborn Screening (NBS) programs (for endocrine, immunologic and metabolic disorders) are effective in reducing child morbidity and mortality. Despite available health services, NBS is not carried out for some newborns. The contributing factors for this should be explored. In high-income settings, homebirth generally refers to planned birth at home, attended by skilled health professionals. We aimed to assess trends and characteristics of planned homebirths and the uptake of NBS and infant health practices. A retrospective case-control study including 3246 infants compared planned homebirth (cases) to age-matched hospital birth controls. During 2016–2023, 0.56% of livebirths (1623/290,458) in the Jerusalem District (JD), Israel, were planned homebirths. The rate has increased since 2020 (COVID-19 pandemic), 0.45% in 2016–2019 vs. 0.67% in 2020–2023. Homebirth infants had a higher birthweight, lower firstborn rate and higher socioeconomic rank. The overall NBS uptake in homebirths was significantly lower (73.7% vs. 99.5% in hospital births) and declined over time (81.1% in 2016–2019 vs. 68.7% in 2020–2023). Regarding preventive health practices for homebirth infants, the registration rate to Mother and Child Health Clinics (MCHCs) was lower (47.1% vs. 92.8% in hospital births), and routine immunization rates were decreased (DTaP-IPV-HiB3 90.7% vs. 60.1%). The NBS uptake among homebirth infants was significantly associated with MCHC registration and routine immunizations (RR = 4.15, 95%CI 3.3–5.3). NBS uptake in homebirths is considerably lower and is associated with subsequent patterns of preventive health practices. Notably, the national NBS program data also indicate a trend of increase in non-uptake rates. Barriers to NBS for homebirths should be identified and targeted interventions implemented. The trends in national NBS non-uptake necessitate further follow-up, and evidence from successful outreach programs should be reviewed and translated into guidelines for health organizations.

## 1. Introduction

Universal Newborn Screening (NBS) programs have become established as fundamental public health measures for reducing child morbidity and mortality [1]. Over the last decades, there have been major technological advances in NBS programs globally [2]. There have also been initiatives aiming to facilitate international cooperation to ensure universal and equal access to NBS [3]. The NBS rates vary markedly across countries and regions and are described as higher in high-income countries [4,5]. Nevertheless, even in settings with highly accessible and affordable health services, NBS uptake rates may vary, and screening is not carried out for some newborns for various reasons [6]. In studies from the US and Canada, the parental choice of planned homebirth has been associated with subsequent inadequate compliance with newborn and infant health recommendations, including NBS, vitamin K prophylaxis at birth, well-child follow-up and routine childhood immunizations [7,8]. Therefore, factors associated with NBS uptake should be explored further.

The global birth cohort of livebirths has been estimated at 134 million in 2022 [9,10]. Throughout human history, childbirth took place at home. During the 20th century, hospital birth became established in high-income countries and, as a gradual process, in low–middle-income countries [11,12,13,14]. An evaluation of childbirth services in 50 countries showed a wide range of homebirth rates, 0.1% to 90% [15]. A Demographic and Health Surveys meta-analysis (67 low–middle-income countries, 2000–2019) showed an overall homebirth rate of 28% [16]; in sub-Saharan Africa, this was a rate of over 50%, in East Asia and the Pacific region, 38%, and in Europe and Central Asia, 5% [16]. Higher rates were linked with inadequate health services, low maternal education, low socioeconomic quintiles and rural dwellings in low–middle income countries [16].

The circumstances in high-income countries are vastly different, and homebirths are generally referred to as planned (elective) births at home, attended by skilled health professionals [11,12,13]. Most European countries reported (2015–2019) homebirth rates of 1% or less [17]. The estimated homebirth rate in the United Kingdom was 2%, in Norway, 3%, in Denmark, 1.4% and in Germany, 1.3%, while the Netherlands reported the highest homebirth rate of 16.3% [17,18]. Data from national US vital statistics reports showed that most of the US out-of-hospital births take place at home [19]. An increase in homebirth rates during the COVID-19 pandemic has been observed and attributed to the awareness of mothers to alternatives to hospital birth [18,19,20,21]. A rise of 20% in out-of-hospital births has been reported between 2019 and 2020 [19]. The US homebirth rate has increased from 1.26% in 2020 to 1.41% in 2021, a 12% increase and the highest level since 1990 [21].

According to the Central Bureau of Statistics in Israel, there are about 180,000 livebirths annually [22]. The vast majority of births take place in hospital maternity wards, with an estimated national planned homebirth rate of less than 0.5% of births [23,24]. The low national homebirth rate in Israel is in line with the majority of the EU countries [17]. A 2018 review on childbirth practices in Israel has postulated that planned homebirths were permitted but not actively encouraged [25]. There is a paucity of data on the characteristics of mothers choosing planned homebirth in Israel as well as on the subsequent utilization of health services for homebirth infants [23,24,26]. The standard recommendations (Ministry of Health, MoH) for the postnatal initial care of newborns at hospitals include physical evaluation and measurements, eye infection prevention, vitamin K prophylaxis, a first dose of Hepatitis B vaccine, a hearing screening and NBS [27]. Similar postnatal recommendations apply for the newborns of planned homebirths [28]. The universal newborn blood-spot screening (for endocrine, metabolic and immunologic disorders) program has been carried out in Israel since 1978, and the range of diseases and conditions included in the screening has been gradually updated [1,5,29]. The screening is offered to all newborns in the hospital setting for hospital births and by attending midwives for homebirths as part of the National Program for the prevention and early detection of birth defects [29,30]. Testing is also available in the Mother and Child Health Clinics (MCHCs). These clinics provide universal preventive health care to infants and pre-school children (aged 0–6 years), free of charge. These clinics are community-based, and the preventive services are widely utilized by the population [31].

The study aims were to describe the rates and trends of planned homebirths, in a large district in Israel, during 2016–2023 (pre, during and post the COVID-19 pandemic), to assess maternal and infant characteristics and to evaluate compliance with the NBS program, registration to MCHCs and routine childhood immunization schedule, among homebirth infants, compared to age-matched hospital births.

## 2. Methods

The definition of homebirth, according to the Ministry of Health (MoH) guidelines in Israel, is a birth taking place at home, by maternal choice, after meticulous planning and preparation [28]. Detailed specific obstetric, environmental and physical requirements are obligatory to ensure both maternal and newborn safety [28]. The MoH guidelines also include the instructions for post-natal care, presented in Supplementary Materials S1 [28]. The National Population Registry Law requires notification of all livebirths (up to 30 days from the date of birth). The hospital administration directly notifies the Population Registry of births. For homebirth notification, a birth attendance statement signed by the attending certified health professional (registered midwife or physician) should be submitted to the local District Health Office, who then report the birth to the Population Registry [28]. Subsequently, all mothers are entitled to receive the National Insurance birth grant [32]. According to the National Newborn Screening Program, the planned homebirth rate was 0.41% in 2022 and 0.48% in 2023 (unpublished data).

The study design was a retrospective, case-control study (planned homebirths compared to hospital births) in the Jerusalem District (JD) during 2016–2023. The district’s population is 1.4 million (2023, 14.7% of Israel’s population). Notably, the JD total livebirths in 2016–2023 (290,458) comprise 20% of the national cohort (1,456,172) [22], which is attributable to the district’s high fertility rates. Data were retrieved from the district’s Newborn Registry, the National NBS database and the National Immunization Registry. The variables included birthplace (homebirth/hospital birth), birthdate, gender, birthweight, birth order, maternal age, education, country of birth, ethnicity and socioeconomic status (ranked place of residence, scale of 1–10). The study period included pre-COVID-19 years 2016–2019, COVID-19 years 2020–2022 and the year 2023. Since the COVID-19 pandemic started at the end of the first quartile of 2020, each year was divided into 4 quartiles (n = 32), and secular trends were observed. The study definition of cases was homebirth infants and matched hospital births as controls. After power calculations, one control per case was randomly selected from the Newborn Registry, matched by the infant’s date of birth. Only infants of singleton births with identification numbers (ID) were included. Case inclusion criteria were infants of planned homebirths (consistent with MoH guidelines) having ID numbers; the birthdate-matched controls were infants having ID numbers and documented hospital births.

The universal NBS (for endocrine, metabolic and immunologic disorders) is included in the health basket of the National Health Insurance Law in Israel [29,30]. The Newborn Screening Program is a national endeavor, and all samples are transferred to, handled and analyzed at a single laboratory. On average, results for 28 conditions are reported on the fourth day of life (Supplementary Materials S2). NBS tests are offered free of charge to all neonates and include parental opt-out consent. The reported NBS compliance rate is high, 99.8% of the national cohort [29]. According to the latest Newborn Screening Program data, overall rates are still high in Israel, 99% in 2022 and 98.6% in 2023 (unpublished data). Generally, blood-spot screening samples are taken at the time of the newborns’ hospital discharge (36 h after birth). For homebirths, midwives are qualified to take samples and are equipped with Guthrie test cards. Blood sampling is also available in the community Mother and Child Health Clinics (MCHCs). According to the guidelines, the test is calibrated to infants’ samples up to four months of age [29].

The National NBS Laboratory receives daily data on all hospital births and once a week data from the Ministry of Interior on new Israeli IDs issued (including homebirths). Cross-filing these data with the NBS samples’ dataset enables a timely identification of newborns who are missing the NBS sample. For newborns with normal NBS results, parents are able to see the screening results on a secure MoH website requiring a personal access code. The District Health Office public health teams receive notifications from the National Laboratory for follow-up of NBS non-uptake as well as on abnormal screening results. For non-uptake cases, the parents are contacted several times by the health teams, and responses are documented. The case definition of “refusers” is applied following three outreach attempts but concluding in non-uptake of NBS.

Preventive health services are provided free of charge to all children (ages birth to 6 years), regardless of civil status, in the community-based MCHC [31]. The routine childhood immunizations are included in the National Health Insurance Law [33,34]. The reported national overall immunization coverage rates are appropriate, with all districts well in line with the World Health Organization (WHO) targets [33,34]. Childhood immunization data are documented in individual digital health records and then incorporated into the National Immunization Registry [33,34].

The study hypothesis was that planned homebirth is associated with compliance to NBS and other infant health practices. According to the MoH guidelines, there are no mandates regarding routine childhood immunizations, and parental consent is required [35,36]. The study design was planned, and the data were analyzed and summarized consistent with the STROBE guidelines (Strengthening the Reporting of Observational Studies in Epidemiology) [37]. The study was approved by the MoH institutional ethical review board and conducted according to applicable MoH guidelines. The study was supported by the Israel National Institute for Health Policy and Health Services Research (NIHP), grant number 2021/50.

Statistical analysis: Data analysis was performed with SPSS version 28.0.0 (IBM, Armonk, NY, USA). Continuous variables were compared by t-test and dichotomous variables by Pearson chi-square test; categorical variables are presented as rate and proportion; continuous variables are presented as mean with standard deviation (SD) and median. Extended Mantel–Haenszel test was used for assessment of trends. Rate ratio (RR), Odds ratio (OR) and 95% confidence intervals (95% CIs) are reported. A logistic regression model was used for planned homebirths and for NBS uptake. Linear regression analysis was used for trends of NBS non-uptake. A *p*-value <0.05 was considered significant for all comparisons.

## 3. Results

**Figure 1:**
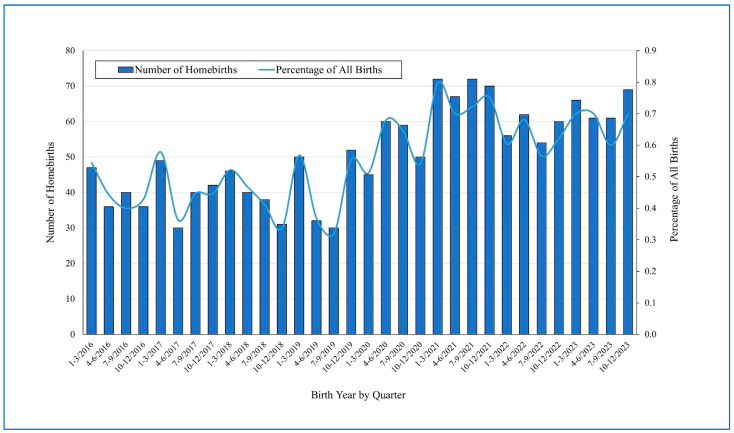
Quarterly numbers of homebirths in the Jerusalem District (32 quarters) in 2016–2023 and the trends of fraction of homebirths all of the district’s livebirths.

During the years 2016–2023, there were 290,458 livebirths in the JD; of these, 1623 were planned homebirths (a mean annual incidence rate of 0.56%). The number of homebirths has increased since 2020 with the COVID-19 pandemic. During the years 2016–2019, 639 homebirths were reported, while 984 were reported during the years 2020–2023 (a 54% rise in the numbers of homebirths). The mean homebirth incidence rate increased from 0.45% during 2016–2019 to 0.66% during 2020–2023, an increase of 46.7% in rates (RR = 1.47, 95%CI 1.33–1.63, *p* < 0.0001). Figure 1 shows the quarterly numbers of homebirths in the district (32 quarters) and the trends of the fraction of all of the district’s livebirths.

The general characteristics of the two groups, the case group (1623 homebirth infants) and the control group (1623 hospital birth infants, matched by birthdate), were compared. The homebirth infants had lower rates of LBW (low birth weight, under 2500 g), showed a higher rate of third and above birth order and a predominance of Jewish ethnicity compared to the age-matched hospital birth controls. Homebirth mothers were older (median age 32 vs. 29 years) and more likely to have been born outside of Israel (mainly in North America) with a higher overall socioeconomic rank (a median rank of the place of residence 5 vs. 2). In a review of the place of residence, several rural medium–high socioeconomic localities showed higher homebirth prevalence rates. The detailed comparison between the cases and controls is presented in Table 1.

A multivariate regression analysis model was employed, following the uni-variable analysis, to evaluate the maternal and child characteristics associated with planned homebirth. The model included the following variables: infant’s gender, birthweight, birth order, maternal country of birth and marital status, place of residence and socioeconomic status. The final model indicated that all of the above-mentioned variables (except infant’s gender) were significantly associated with planned homebirth. The explained variation of the multivariate regression model was 24.1%.

**Figure 2:**
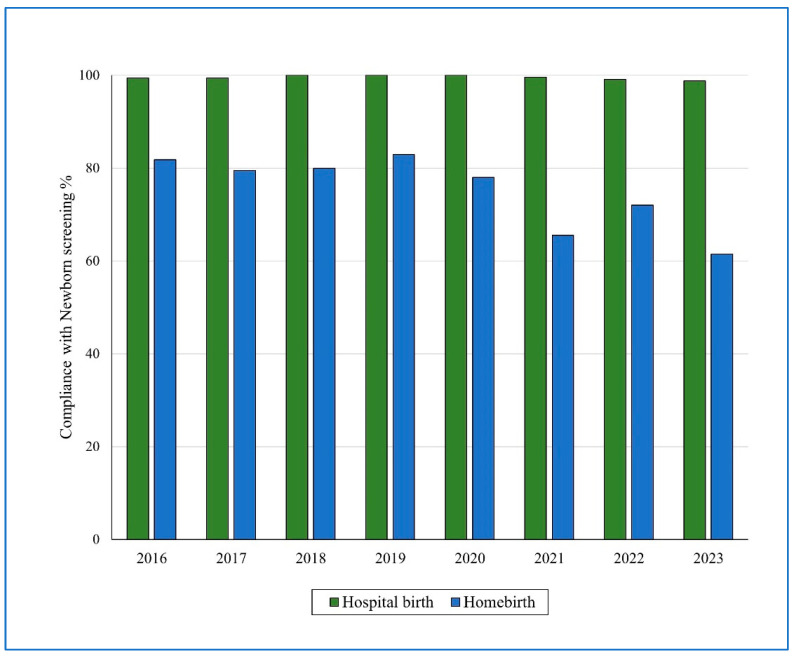
Annual rates of newborn screening uptake in homebirth infants and hospital birth infants, 2016–2023.

The overall newborn screening rate was significantly lower in the homebirth infant group compared to the group of hospital birth infants. Of all the 3246 infants included in the study, 2809 infants underwent NBS. Among the cases, 73.6% (1194/1326) underwent NBS and among the controls 99.5% (1615/1623). While the vast majority of hospital birth infants underwent NBS, over a quarter of homebirth infants showed non-uptake (RR = 1.35 95%CI 1.3–1.39, *p* = 0.0001). NBS coverage rates among the homebirth infants declined significantly over time, from 81.1% during the years 2016–2019 to 68.7% during the years 2020–2023 (RR = 1.18 95%CI 1.12–1.25, *p* = 0.0001). In comparison, the NBS rates among the hospital birth infants declined slightly from 99.7% (in 2016–2019) to 99.4% (in 2020–2023). The lowest NBS rates were observed in 2023 (the last year of the study), 98.8% among hospital birth infants and 61.5% among homebirth infants. The annual rates of NBS uptake in the two study groups, homebirth infant group compared hospital birth infants, are presented in Figure 2.

Of all of the 3246 infants in the study population, 2273 infants were registered to the community Mother and Child Health Clinic (MCHC), 766/1623, 47.2% of the cases compared to 1507/1623, 92.9% of the controls. The MCHC registration rate was significantly lower in homebirth infants (RR = 1.97 95%CI 1.87–2.07, *p* = 0.0001). The MCHC registration rate in the case group had declined considerably from 56.2% during 2016–2019 to 41% during 2020–2023 (RR = 1.36, 95%CI 1.24–1.51, *p* = 0.0001). Notably, the MCHC registration rate also showed some decline among the control group from 96.6% in 2016–2019 to 90.4% in 2020–2023 (RR = 1.07, 95%CI 1.04–1.09, *p* = 0.0001).

As the NBS uptake rates were very high in the hospital birth infants, we implemented a sub-group analysis on the planned homebirth infant group. The NBS had not been performed in about a quarter of homebirth infants (429/1623, 26.4%). The sub-groups differed as to gender, birth weight, birth order, mother’s birth country, marital status, education, previous homebirth, place of residence and socioeconomic status. The comparison between the sub-groups is presented in Table 2.

**Figure 3:**
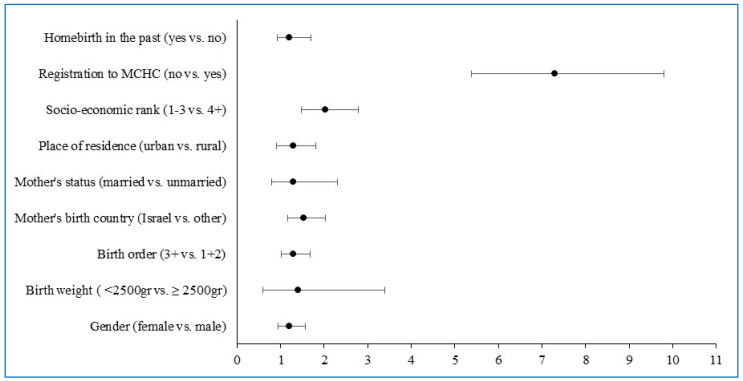
Odds ratio (OR) for non-uptake of newborn screening among planned homebirth infants—multiple logistic regression model results.

A multivariate regression analysis model was utilized to evaluate the maternal and child characteristics associated with NBS performance among the planned homebirth infants. The final model indicated that child birth order, mother’s birth country and socioeconomic status were significantly associated with NBS. Registration to the MCHC was strongly associated with the uptake of NBS (Figure 3).

**Figure 4:**
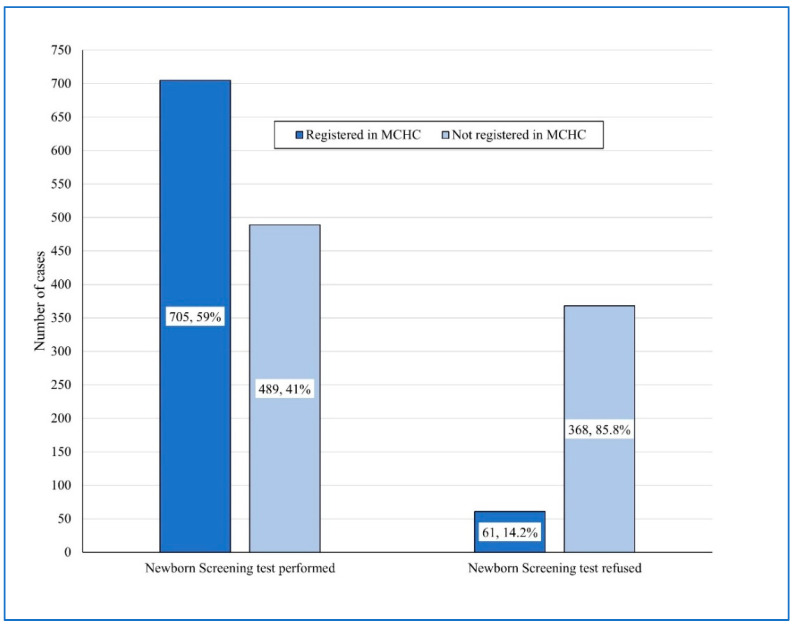
Association between newborn screening uptake and Mother and Child Health Clinic registration among the homebirth infants.

The association between the uptake of NBS and the MCHC registration in the homebirth infant group was assessed (Figure 4). The homebirth infants who did not undergo NBS were also significantly less likely to be registered to the MCHC compared to the homebirth infants who had undergone NBS (14.2% vs. 59% MCHC registration, RR = 4.15, 95%CI 3.3–5.3, *p* = 0.001).

Routine immunizations rates were evaluated in infants born in 2016–2022 (*n* = 1372 in each group). The coverage rates for the vaccines HBV1, HBV3, DTaP-IPV-HiB3 and MMR1 were 96%, 86.2%, 90.7% and 90.1%, respectively, among hospital birth infants, registered to the MCHC. Of the homebirth infants registered to the MCHC, the coverage rates were considerably lower (53.7%, 38.1%, 60.1% and 62.6%, respectively). When including all of the participants in the homebirth infant group, the immunization rates for the vaccines DTaP-IPV-HiB3 and MMR1 were very low 28.5% and 29.7%. We also compared the coverage rates in the homebirth infants registered to the MCHC divided according to the NBS performance. The coverage rates for the vaccines DTaP-IPV-HiB3 and MMR1 were significantly lower in infants without NBS, 34.1% and 43.9%, respectively, compared to 71.7% and 63.9% among infants with NBS performance (for the DTaP-IPV-HiB3, RR = 2.1, 95%CI 1.37–3.2, *p* = 0.0001 and, for the MMR1, RR = 1.45, 95%CI 1.02–2.07, *p* = 0.009).

Data on newborn vitamin K acceptance, refusal and mode (intramuscular injection or oral drops) were retrieved from the medical records (where available). Overall, the compliance with vitamin K was decreased in homebirth infants who had not undergone NBS (45%) compared to homebirth infants who had undergone NBS (90.8%). Most homebirth infants who had not undergone NBS (80%) received the oral drops, while most homebirth infants who had undergone NBS received the vitamin K intramuscular injection (70.7%). By comparison, amongst the hospital birth infants, 99.5% received the vitamin K intramuscular injection.

**Figure 5:**
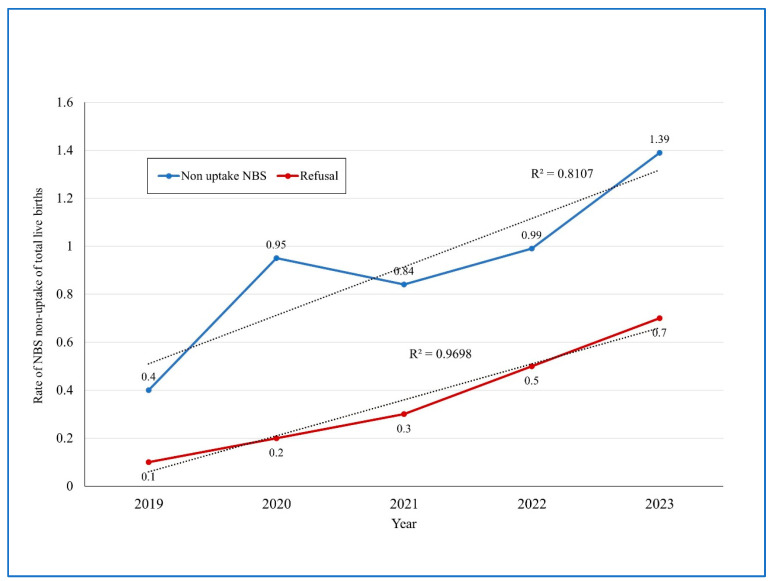
National data on yearly newborn screening non-uptake rates 2019–2023, pre (upper curve) and post-outreach (lower curve).

Aiming for additional assessment, we also extracted summarized data from the National NBS program annual reports, including all hospital births in the country. During the years 2019–2023, the overall primary rate of NBS non-uptake was 0.92% nationally (8297 infants of 906,749). After the comprehensive outreach process performed by the community public health teams, the overall NBS non-uptake rate declined significantly to an 0.4% of all infants (RR =2.5, 95%CI 2.37–2.56, *p* = 0.0001). Notably, the rates of NBS non-uptake nationally increased considerably between the years 2019 and 2023. Figure 5 shows the rising trend of non-uptake and refusal regarding NBS nationally over the years 2019–2023, the reduction in NBS non-uptake after the comprehensive outreach process and the two linear regression lines (r^2^ = 0.81 and 0.96, respectively).

## 4. Discussion

The current study on planned homebirths included 3246 infants in a retrospective case-control study design (1:1 ratio). The characteristics of homebirth and hospital birth mothers and infants differed. Homebirth infants had a higher birth weight, a lower rate of firstborns and a higher Jewish ethnicity rate. Homebirth mothers were older and more likely non-Israeli born (mainly North American) with higher socioeconomic rank.

The study findings indicated that the overall uptake rates of NBS (for endocrine, immunologic and metabolic disorders) in homebirth infants were significantly lower (73.7% compared to 99.5% in hospital births) and declined over time (81.1% uptake in 2016–2019 and 68.7% in 2020–2023). Regarding the preventive health practices among homebirth infants, the registration rate to the MCHC was lower (47.1% vs. 92.8%), routine immunization rates were decreased and the acceptance rate of vitamin K at birth was lower. The NBS uptake among the homebirth infants was significantly associated (RR = 4.15) with subsequent registration to the MCHC and the acceptance of routine childhood immunizations.

The findings on decreased NBS uptake are notable since there was a marked increase in the homebirth rate in our study since 2020 (the emergence of the COVID-19 global pandemic). The homebirth rate increased from 0.45% of all livebirths in 2016–2019 to 0.67% in 2020–2023. Similar trends of increase in the homebirth rates have been reported in several studies from the US, Canada and Europe [17,19,20,21,38,39].

In a large US study of 40,440 births, 94% of NBS refusals were associated with homebirths [7]. A non-uptake of NBS can lead to a risk of a late diagnosis of an infant with a congenital endocrine, metabolic or immunologic disorder. Early appropriate treatment can prevent and reduce symptoms, thereby contributing to survival and the quality of life. Childhood immunizations reduce morbidity and mortality from infectious diseases of the infant and also of other children and adults with whom he/she comes into contact. Other studies showed that although vitamin K refusal is uncommon, those who refuse are more likely to have given birth at home or in a birth center. Homebirth infants are also less likely to accept the recommended routine childhood immunizations [8,40,41]. In a Canadian study of a cohort of 282,378 infants, planned homebirths showed a vaccine refusal rate of 14.5% compared to 0.2% of hospital births. This study also showed that, overall, the risk of being completely unimmunized with any of the scheduled childhood vaccines at age 15 months was 14.6 times higher in those refusing vitamin K compared to those receiving it [8]. Vitamin K “Hesitancy” has also been identified and is associated with childhood immunization hesitancy [42], also referred to as Vaccine Hesitancy [34]. A study in the US has showed that planned homebirth is a risk factor for a non-receipt of the HBV vaccine birth dose, with 99.5% non-receipt in homebirths compared to 23.7% of hospital births [43].

The data from the national screening program regarding hospital births also show a trend of increase in the NBS non-uptake rate from 0.4% in 2019 to 1.39% in 2023. The outreach efforts performed by the public health teams can be successful in the encouragement of parents to accept NBS for their infants. Thus, in 2023, the non-uptake rate was reduced, after outreach, from 1.39% to 0.7% of hospital births. The outreach efforts include telephone calls and home visits by qualified public health nurses. In Israel, home-visit nurses from the district health office work in tandem with community MCHC nurses. It is important for health organizations to be ready to provide more home visits for this group in the event that the homebirth trend further increases and, associated with it, more non-uptake of NBS. In low- and middle-income countries, home visits by trained community health workers may successfully identify newborns and young infants (up to 59 days of age) with serious illnesses and improve care-seeking from a health facility [44]. There are noticeable variations in NBS practice in different countries, even with nationally established guidelines, including information given to parents about screening and whether NBS is encouraged as an opt-in or opt-out procedure [6]. Therefore, barriers to NBS for homebirth mothers should be investigated and identified and targeted interventions implemented. Trends in national NBS non-uptake should be followed and evidence from successful interventions reviewed and translated into outreach work protocols for public health teams.

The American Academy of Pediatrics (AAP) does not indorse planned homebirth. However, the AAP recognizes that women may choose to plan a homebirth and has compiled a policy document, with essential elements of care for the healthy-term newborn infants born at home [45]. The low national homebirth rate in Israel is more in line with the rates in the majority of the EU countries [17]. A review in 2018 postulated that homebirth in Israel was not actively encouraged. The authors concluded that public health emphasis appeared to be on the prevention of disease in newborns by timely screening and that the hospital setting was considered by the medical establishment to be far more conducive to this [25].

However, the increase in homebirth rates should not be overlooked. A recent Cochrane review comparing planned hospital births with planned homebirths for women at a low risk of complications concluded that a legislated and superintended homebirth system as an alternative to hospital birth is essential [46]. Hence, physicians and nurses should be equipped to support their patients’ decision-making by presenting clear, balanced information about homebirths [47]. Recently, the MoH in Israel published guidelines for establishing community birth centers [48]. These centers offer person-centered care, are staffed by midwives and obstetricians and are located near a hospital should advanced care be required. The newborn health guidelines are similar to those in hospital births including NBS [27].

The complexity of NBS programs and the ongoing increase in the number of conditions on the screening panels present a public health challenge to guarantee integrated care and program effectiveness [49]. In striving to contribute towards improved health outcomes, research on health service utilization and barriers to compliance with infant health recommendations is essential. International cooperation is advancing to facilitate universal access to NBS [3]. New NBS technologies are emerging, including genomic testing, and it has been suggested that the public should be included in discussions on how these technologies should be implemented so that concerns can be addressed [3]. A study from Germany noted the essentiality of well-coordinated NBS programs with continuous quality assessment and special attention to the perspectives of parents and families [50]. A study from Thailand showed that about 60% of parents were defined as having appropriate NBS awareness, while only 10% of them had good knowledge [51]. Hence, NBS guidance (for both parents) should preferably be initiated during antenatal care [51]. In a systematic review on screening in public health and clinical care, the recommendation was that public health screening programs should be developed with a strong focus on consistency in terminology and clarity in definition [52]. Our field experience shows that specifically the term NBS may lead to misinterpretation (“screening” in Hebrew is the same word as “survey”, which may imply mass information gathering). Working with the public to describe NBS in lay terms as well as providing culturally tailored and easily accessible information is, therefore, crucial.

The current research findings underpin the need to work together with health care professionals involved in homebirths as others have pointed out [23]. Midwives and physicians attending homebirths are key players in providing explanations to parents and promoting the acceptance of NBS. They should be involved in defining what information parents should receive [53].

Health professionals in the community and in hospitals have a crucial role in promoting the acceptance of the recommended care for newborns and infants. Pediatricians should have access to the data showing newborns who have had and have not had NBS so that they can explain the results and encourage uptake.

Further research is required to understand both the barriers to NBS amongst homebirths parents and the attitudes and behavior of homebirth-attending midwives. The National Midwives Association should be a partner in this research. The results of this research should inform targeted interventions.

The overall rate of NBS acceptance in our study was higher than routine immunization coverage rates. NBS is defined as a secondary preventive intervention, and childhood immunizations are defined as a primary preventive intervention. While NBS involves blood-spot sampling, immunizations involve the acceptance of antigens and adjuvants included in the vaccine. Further research is required to compare the reasons for NBS refusal and immunization refusal and the interrelationships between different affecting factors.

### Limitations

The current study is subject to several limitations. It is an observational retrospective study, including all notified planned homebirths in a well-defined geographical area (the JD) compared to hospital births. Consequently, it might be challenging to make a generalization of the study findings. The distinctive socio-demographic factors, age distribution, living conditions, household size, service utilization patterns and attributes of health facilities, which significantly affect NBS uptake, may differ extensively between various settings and population groups, making comparisons difficult. The local public health infrastructure and outreach capabilities, as well as the national NBS laboratory capacity, may also differ considerably between settings.

## 5. Conclusions

NBS uptake in homebirths is considerably lower and is associated with subsequent patterns of preventive health practices. Notably, the national NBS program data also indicate a trend of decrease in uptake rates. Barriers to NBS for homebirths should be identified and targeted interventions implemented. The trends in national NBS non-uptake necessitate further follow-up, and evidence from successful outreach programs should be reviewed and translated into guidelines for health organizations.

## Figures and Tables

**Table 1:** General characteristics of the case group (homebirth infants, *n* = 1623) and the control group (hospital births matched by birthdate).

Variable	Study Group *n* = 1623	Control Group *n* = 1623	OR (95%CI)	*p*-Value
**Gender**				
Male	806 (49.7%)	826 (50.9%)	0.95 (0.83–1.09)	NS
Female	817 (50.3%)	797 (49.1%)		
**Birth Weight**				
<2500 gr	28 (1.8%)	86 (5.3%)	0.31 (0.2–0.49)	0.001
Birth weight (gr) mean ± SD	3335 ± 444	3288 ± 499		0.006
Birth weight median (gr)	3330	3300		NS
**Birth order**				
1 and 2	872 (53.7%)	965 (59.5%)		
3+	751 (46.3%)	658 (40.5%)	1.26 (1.1–1.46)	0.001
**Mother’s age (years)**				
Mean ± SD	31.6 ± 5.6	29.5 ± 5.9		0.001
Median	32	29		0.001
**Mother’s birth country**				
Israel	1087 (67%)	1401 (86.3%)	0.3 (0.26–0.38)	0.0001
Other	536 (33%)	222 (13.7%)		
**Mother’s status**				
Unmarried	153 (9.4%)	28 (1.7%)	5.93 (3.9–9.3)	0.0001
Married	1470 (90.6%)	1595 (98.3%)		
**Place of residence**				
Rural	544 (33.5%)	155 (9.6%)	4.77 (3.9–5.84)	0.0001
Urban	1079 (66.5%)	1468 (90.4%)		
**Maternal education (years)**				
Mean ± SD	14.7 ± 2.4	13.6 ± 2.1		0.001
Median	15	12		0.001
**Ethnicity**				
Jewish	1623 (100%)	1332 (82.1%)		0.0001
Arab	0 (0%)	291 (17.9%)		
**Socio-economic rank of place of residence**				
1–3	704 (43.4%)	1169 (72%)		
4+	919 (56.6%)	454 (28%)	3.36 (2.89–3.9)	0.0001
Mean ± SD	4.3 ± 2.5	2.8 ± 2.1		0.001
Median	5	2		0.001
**Homebirth in the past**				
Yes	772 (47.6%)	-------		0.0001
No	851 (52.4%)	1623 (100%)		

**Table 2:** NBS rate comparison between the sub-groups of the homebirth cases.

		Performed Newborn Screening Test			
Variable	Total	No	Yes	OR (95%CI)	*p*-Value
		*n* = 429 (26.4%)	*n* = 1194 (73.6%)		
**Gender**					
Male	806	193 (45%)	613 (51.3%)	0.78 (0.62–0.97)	0.014
Female	817	236 (55%)	581 (48.7%)		
**Birth weight**					
<2500 gr	28	12 (2.8%)	16 (1.4%)	2.08 (0.89–4.73)	0.047
Birth weight (gr) mean ± SD	3335 ± 444	3284 ± 443	3353 ± 444		0.006
Birth weight median (gr)	3330	3300	3350		NS
**Birth order**					
1 and 2	872	205 (47.8%)	667 (55.9%)	0.72 (0.58–0.91)	0.002
3+	751	224 (52.2%)	527 (44.1%)		
**Mother’s age (years)**					
Mean ± SD	31.6 ± 5.6	31.2 ± 6	31.7 ± 5.5		NS
Median	32	32	32		NS
**Mother’s birth country**					
Israel	1087	316 (73.7%)	771 (64.6%)	1.5 (1.19–1.98)	0.0001
other	536	113 (26.3%)	423 (35.4%)		
**Mother’s status**					
Unmarried	153	21 (4.9%)	132 (11.1%)	0.41 (0.24–0.67)	0.0001
Married	1470	408 (95.1%)	1062 (88.9%)		
**Homebirth in the past**					
Yes	772	231 (53.8%)	541 (45.3%)	1.4 (1.12–177)	0.001
No	851	198 (46.2%)	653 (54.7%)		
**Place of residence**					
Rural	544	90 (21%)	454 (38%)	0.43 (0.33–0.56)	0.0001
Urban	1079	339 (79%)	740 (62%)		
**Maternal education (years)**					
Mean ± SD	14.6 ± 2.4	13.6 ± 1.9	15.04 ± 2.4		<0.001
Median	15	12	15		<0.001
**Socio-economic rank of place of residence**					
1–3	704	271 (63.2%)	433 (36.3%)	3.01 (2.38–3.82)	0.0001
4+	919	158 (36.8%)	761 (63.7%)		
Mean ± SD	4.3 ± 2.5	3.2 ± 2.4	4.7 ± 2.4		<0.001
Median	5	2	6		<0.001

## Data Availability

Data are available at the researchers’ database.

## Supplementary Materials

The following supporting information can be downloaded at: https://www.mdpi.com/article/10.3390/ijns11010015/s1, Supplementary Materials S1: MoH Homebirth Newborn Treatment Guidelines (Section 6); Supplementary Materials S2: MoH NBS—list of 28 conditions.

## Abbreviations

NBS	Newborn Screening
MCHC	Mother and Child Health Clinics
JD	Jerusalem District
MoH	Ministry of Health
ID	Identification Number
WHO	World Health Organization
STROBE	Strengthening Reporting of Observational Studies in Epidemiology
NIHP	Israel National Institute for Health Policy and Health Services Research
