# Supplementary material for: Non-Uptake of Newborn Screening in Planned Homebirth Is Associated with Preventive Health Practices for Infants: A Retrospective Case-Control Study
Source: Int J Neonatal Screen. 2025 Feb 21;11(1):15. doi: 10.3390/ijns11010015 (PMC11942714; doi:10.3390/ijns11010015)
Supplement: Supplementary file 1 [file IJNS-11-00015-s001.zip › IJNS-3473570-supplementary.pdf]

SUPPLEMENT S1: MoH Homebirth Newborn Treatment Guidelines (Section 6)

The MoH respects the right and freedom of every woman to choose where to give birth. However, it also sees as its professional and moral duty, to emphasize that birth in recognized and licensed maternity wards is safer for both mother and infant. Nevertheless, since homebirths take place in Israel, the following guidelines apply, in order to establish a balance between the mother's freedom of choice and the need to maintain the safety of the mother and of the newborn (who does not have freedom of choice).

For a homebirth, the following treatment guidelines apply if the mother does not wish to go to hospital within the first 24 h after the birth: preventive eye treatment for the newborn; newborn vitamin K injection soon after birth or 3 doses of oral vitamin K at set times; if the mother is Rh-negative and has previously had blood types antigen antibodies in the blood serum, blood typing of the newborn, hemoglobin and bilirubin; when the mother is Rh-negative and the newborn Rh-positive, anti D injection for the mother; newborn blood sampling for metabolic diseases as per hospital born (see supplement 2), at the earliest 48 h after birth and not later than 7 days; hearing test. In addition, the mother should receive information and guidance on the following topics: breastfeeding; feeding; sleeping (positions); nappy changing; washing; cleaning excretions; care of the navel; clothing; seasonal room temperatures; car seat safety; the need for a pediatrician checkup within 24 h of birth and thereafter MCHC or pediatrician follow up; the need for Hepatitis B (HBV) immunization for the newborn at an MCHC within 24 h of birth; referral to the Interior Ministry to register the newborn.

SUPPLEMENT S2: MoH National Newborn Screening Program for 28 disorders

1. Classical phenylketonuria (PKU)
2. Argininosuccinic aciduria (ASAL)
3. Citrullinaemia type I (ASAS)
4. Hyperornithinemia, hyperammonemia and homocitrullinuria syndrome with severe neonatal hyperammonemia (HHH)
5. Classical homocystinuria (CBS deficiency)
6. Maple Syrup Urine Disease (MSUD)
7. Ornithine transcarbamylase deficiency with severe neonatal hyperammonemia (OTC)
8. Tyrosinaemia type 1 (TYR-I)
9. 3-Hydroxy-3-methylglutaric aciduria (3HMG)
10. Beta-ketothiolase deficiency (BKT)
11. Glutaric acidaemia type I (GA I)
12. Isovaleric acidaemia (IVA)
13. Isolated methylmalonic acidaemia (MMA, mut^{0}, mut^{-})
14. Multiple carboxylase deficiency (MCD, MADD)
15. Propionic acidaemia (PA)
16. Carnitine palmitoyltransferase deficiency type I (CPT-I)
17. Carnitine palmitoyltransferase type II neonatal onset (CPT-II)
18. Carnitine-acylcarnitine translocase deficiency (CACT)
19. Medium-chain acyl-CoA dehydrogenase deficiency (MCAD)
20. Very long-chain acyl-CoA dehydrogenase deficiency (VLCAD)
21. Long-chain L-3-hydroxyacyl-CoA dehydrogenase deficiency (LCHAD)
22. Trifunctional protein deficiency (TFP)
23. Methylenetetrahydrofolate reductase (MTHFR)
24. Cobalamin C deficiency (Cbl-C)
25. Classical galactosaemia (GALT)
26. Congenital adrenal hyperplasia - salt wasting, 21 hydroxylase deficiency (CAH)
27. Primary congenital hypothyroidism (CHT)
28. Severe combined immunodeficiency (SCID)
